# Persistent Anemia in the Setting of a Rare Pancreatic Pseudoaneurysm

**DOI:** 10.1155/2021/5550005

**Published:** 2021-05-05

**Authors:** Olivia Peralta, Christopher Chew, Matthew Newcomb

**Affiliations:** ^1^American University of the Caribbean School of Medicine, Cupecoy, Saint Martin; ^2^Department of Graduate Medical Education, Northeast Georgia Health System, Gainesville, GA, USA; ^3^Department of Hospital Medicine, Northeast Georgia Health System, Gainesville, GA, USA

## Abstract

*Introduction*. Pancreatic pseudoaneurysm is a rare but potentially fatal complication that can follow pancreatitis. While early detection is critical to preventing poor long-term outcomes, clinical features vary significantly. Most often, abdominal pain is the presenting complaint, but this can be complicated as classic symptoms of pancreatitis also present with abdominal pain. Herein, we present a patient with an acute on chronic gastrointestinal bleed that was finally attributed to a pancreatic pseudoaneurysm. *Case Presentation.* The patient was a 56-year-old male with a past medical history significant for epilepsy, alcohol abuse, and hypertension who presented as a transfer from an outside facility for a gastrointestinal bleed. Prior to presentation, the patient reported rectal bleeding over the prior 1.5 months but had not sought care until bleeding increased along with increased abdominal pain. The patient's hemoglobin was 6.3 at presentation of the outside facility and received a total of four units of packed red blood cells (PRBCs) prior to arrival. After arrival, persistent bleeding was noted, and an additional 2 units of PRBCs were transfused. A computed tomography angiography (CTA) of the abdomen was obtained to identify the source for embolization. This, however, revealed a 4 × 4 × 3.5 cm intrinsically dense or enhanced mass of the pancreatic head. *Discussion.* Pancreatic pseudoaneurysm is a rare but potentially fatal complication that can follow pancreatitis. In chronic pancreatitis patients who underwent imaging incidence is estimated to be up to 10%. Treatment is difficult, and coil embolization is often used, though this can lead to splenectomy due to splenic ischemia. Stent grafts can be used in the surrounding arteries to maintain the integrity of viscera but carry risk of stent-related thrombosis. Further research is needed on the optimal management of this potentially lethal complication of pancreatitis.

## 1. Introduction

Pancreatic pseudoaneurysm is a rare but potentially fatal complication that can follow pancreatitis. Thought to be due to an erosion of vessels by pancreatic enzymes, aneurysms most often involve the splenic artery but can affect other branches [1]. Approximately 10% of chronic pancreatitis patients who undergo imaging are found to have developed pseudoaneurysms, with many of these developing within pseudocysts or forming fistulas with surrounding structures [1]. Due to their complexity, it is thought that these pseudoaneurysms can take from weeks to even years to form [2]. While early detection is critical to preventing poor long-term outcomes, clinical features vary significantly. Most often, abdominal pain is the presenting complaint, but this can be complicated as classic symptoms of pancreatitis also present with abdominal pain [3]. Herein, we present a patient with an acute on chronic gastrointestinal bleed that was finally attributed to a pancreatic pseudoaneurysm.

## 2. Case Report

**Figure 1:**
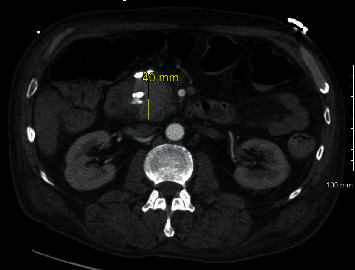
Computed tomography with angiography with measurement of pseudoaneurysm.

**Figure 2:**
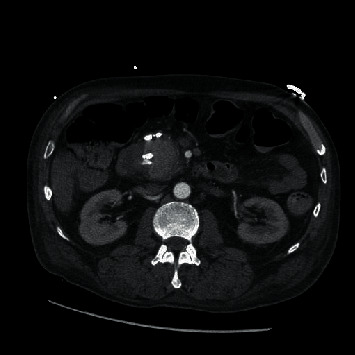
Computer tomography without measurements of pseudoaneurysm.

**Figure 3:**
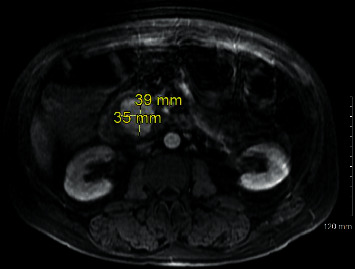
Magnetic resonance imaging with measurement of pseudoaneurysm.

**Figure 4:**
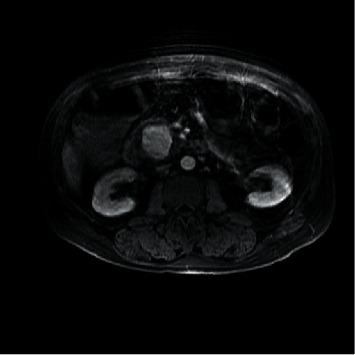
Magnetic resonance imaging without measurement of pseudoaneurysm.

The patient was a 56-year-old male with a past medical history significant for epilepsy, alcohol abuse, and hypertension who presented as a transfer from an outside facility for a gastrointestinal bleed. Prior to presentation, the patient reported rectal bleeding over the prior 1.5 months but had not sought care until bleeding increased along with increased abdominal pain. The patient's hemoglobin was 6.3 g/dL (normal range: 13.5–17.5 g/dL for men) at presentation of the outside facility and received a total of four units of packed red blood cells (PRBCs) prior to arrival due to continued bleeding. Upon arrival to our facility, the patient was experiencing alcohol withdrawals requiring ongoing management with lorazepam and dexmedetomidine. Upon arrival, there was initial moderate abdominal pain that was diffuse. The patient was started on intravenous (IV) pantoprazole twice daily along with hemoglobin monitoring. Gastroenterology performed an esophagogastroduodenoscopy (EGD) on hospital day one that did not identify a source of bleeding. With continued bleeding, a tagged red blood cell (tRBC) scan was performed which showed an upper gastrointestinal (GI) source of bleeding, likely in the distal stomach or proximal duodenum. With persistent bleeding and having received a total of 9 units of PRBCs at this time, a computed tomography angiography (CTA) of the abdomen was obtained to identify the source for potential embolization. This, however, revealed a 4 × 4 × 3.5 cm intrinsically dense or enhanced mass of the pancreatic head, as shown in Figures 1 and 2. Magnetic resonance cholangiopancreatography (MRCP) was obtained which showed findings suggestive of pseudoaneurysm involving the pancreatic head, as shown in Figures 3 and 4.

**Figure 5:**
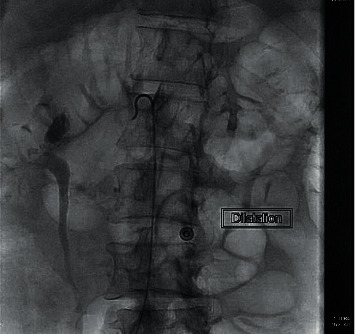
Dilatation evident during procedure.

**Figure 6:**
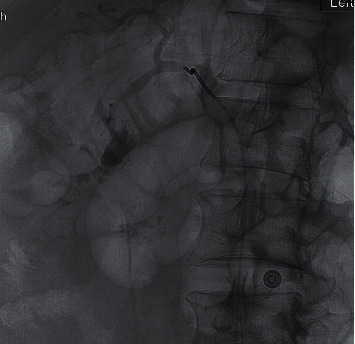
Contrast-enhanced image during the embolization.

**Figure 7:**
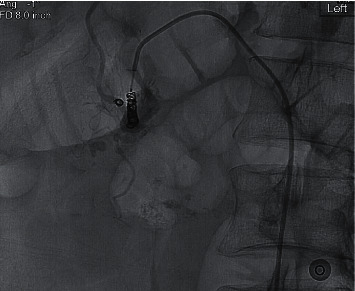
Multiple coils delivered at the side of the pseudoaneurysm during embolization.

Vascular surgery was consulted, and percutaneous embolization of the pseudoaneurysm was attempted as shown in Figures 5 and 6. Specifically, a catheter was inserted into the common femoral artery with ultrasound guidance, and it was then guided into the superior mesenteric artery and celiac axis and trunk. Selective injection of contrast was performed there, and then it was guided further into the gastroduodenal artery where a microcatheter was positioned at the distal end, corresponding to the location noted in the CT scan. Four 3 to 5 mm diameter coils were delivered, as shown in Figure 7, and the procedure ended. Bleeding failed to improve after the procedure, likely due to the sheer size and length of time it had been developing. After discussion with the general surgery team and consultation with an outside hepatobiliary surgeon, the patient was then transferred to a tertiary care center for surgical intervention.

When he arrived at the tertiary care center, he was monitored for seven days to evaluate for resolution since embolization was attempted immediately prior to transfer. After there was no resolution, he was taken to the operating room for an open Whipple procedure, cholecystectomy, and repair of a portal vein. All removed specimens were sent for surgical pathology evaluation. The gall bladder showed chronic cholecystitis, and pancreatic slices for the frozen section showed chronic pancreatitis with a dilated pancreatic duct negative for high-grade dysplasia or malignancy. The Whipple procedure yielded a bile duct, small intestine, and stomach that were all unremarkable and negative for carcinomas, as well as surgical margins and 31 lymph nodes that were also negative for carcinomas. The pancreas removed during the Whipple showed intraductal papillary mucinous neoplasm with focal high-grade dysplasia in the main duct, as well as chronic pancreatitis. Postoperatively, the patient did well and only required one week of monitoring before discharge. At the time of discharge, he was ambulating, having bowel movements, eating a normal diet, and had all drains removed. His pain was controlled with oral medications, and he had negative blood cultures. He was instructed to follow-up with his primary care physician and hepatobiliary surgeon within two weeks of discharge.

## 3. Discussion

Pancreatic pseudoaneurysm is a rare but potentially fatal complication that can follow pancreatitis. In chronic pancreatitis patients who underwent imaging, incidence is estimated to be up to 10%, and notably, when associated with a concurrent pseudocyst, the bleeding risk is estimated at 15–20% [1]. While difficult to diagnose due to vague presenting symptoms, early localization and identification is important. Since chronic pancreatitis is a known risk factor for pseudoaneurysms, it is important to follow-up with these patients closely. However, as the clinical presentation of chronic pancreatitis can be vastly different, there is difficulty in following these patients on a regular basis. The American Pancreatic Association released guidelines in 2014 which offer direction in management of the patient with chronic pancreatitis [4]. Primarily, when chronic pancreatitis is clinically suspected, a contrast-enhanced CT is recommended along with lifestyle modifications such as the cessation of smoking and alcohol intake, as well as a low-fat diet [4]. Pain is the most common symptom that accompanies chronic pancreatitis, and thus, it is vital to manage the pain appropriately [5]. When symptoms of malabsorption or steatorrhea appear, it may be indicated to initiate pancreatic enzyme replacement [6]. If strictures, stones, or pseudocysts arise and are symptomatic, they can often be treated with endoscopy or if larger disease is present within the ducts, decompressive surgery such as a Whipple procedure might be indicated [6]. Often, pseudoaneurysms can manifest as asymptomatic anemia with or without melena, or silent bleeding into the peritoneal cavity [7].

While pancreatic pseudocysts resolve spontaneously in up to 60% of cases, it is important to rule out underlying pseudoaneurysms [8]. Small pseudoaneurysms can go undetected, but angiography is the gold standard for identifying both bleeding pseudoaneurysms as well as pseudocysts [9]. In 1976, White et al. identified the importance of differentiating between pseudoaneurysms and carcinoma of the pancreas in which unnecessary clinical biopsy can often worsen the progression if it is not a carcinoma [10]. In a study conducted by Zabicki, et al, the splenic artery was ruled as the most common site of pseudoaneurysm, followed by the hepatic artery and then right gastroepiploic artery [11].

Various endovascular procedures are used in the management of pseudoaneurysms, depending upon the hemodynamic stability of the patient as well as the vessel involved [11]. Coil embolization is often used, though this can lead to splenectomy due to splenic ischemia. Stent grafts can be used in surrounding arteries to maintain the integrity of viscera but carry risk of stent-related thrombosis. Thrombin injections, or thrombin-related injections, with ultrasound guidance can be used when primary involved vessels cannot be easily identified [11]. Further research is needed on the optimal management of this potentially lethal complication of pancreatitis.
